# Discovering Molecules That Regulate Efferocytosis Using Primary Human Macrophages and High Content Imaging

**DOI:** 10.1371/journal.pone.0145078

**Published:** 2015-12-16

**Authors:** Sandra Santulli-Marotto, Alexis Gervais, Jamie Fisher, Brandy Strake, Carol Anne Ogden, Chelsea Riveley, Jill Giles-Komar

**Affiliations:** 1 Janssen Research & Development, LLC, 1400 Welsh & McKean Rds., Spring House, PA, 19477, United States of America; 2 FlowMetric, Inc., 3805 Old Easton Road, Doylestown, PA, 18902, United States of America; The Hospital for Sick Children and The University of Toronto, CANADA

## Abstract

Defective clearance of apoptotic cells can result in sustained inflammation and subsequent autoimmunity. Macrophages, the “professional phagocyte” of the body, are responsible for efficient, non-phlogistic, apoptotic cell clearance. Controlling phagocytosis of apoptotic cells by macrophages is an attractive therapeutic opportunity to ameliorate inflammation. Using high content imaging, we have developed a system for evaluating the effects of antibody treatment on apoptotic cell uptake in primary human macrophages by comparing the Phagocytic Index (PI) for each antibody. Herein we demonstrate the feasibility of evaluating a panel of antibodies of unknown specificities obtained by immunization of mice with primary human macrophages and show that they can be distinguished based on individual PI measurements. In this study ~50% of antibodies obtained enhance phagocytosis of apoptotic cells while approximately 5% of the antibodies in the panel exhibit some inhibition. Though the specificities of the majority of antibodies are unknown, two of the antibodies that improved apoptotic cell uptake recognize recombinant MerTK; a receptor known to function in this capacity *in vivo*. The agonistic impact of these antibodies on efferocytosis could be demonstrated without addition of either of the MerTK ligands, Gas6 or ProS. These results validate applying the mechanism of this fundamental biological process as a means for identification of modulators that could potentially serve as therapeutics. This strategy for interrogating macrophages to discover molecules regulating apoptotic cell uptake is not limited by access to purified protein thereby increasing the possibility of finding novel apoptotic cell uptake pathways.

## Introduction

Phagocytes, such as macrophages, are largely responsible for phagocytosis of apoptotic cells, or efferocytosis [1], and impairments in this process have been proposed as a potential mechanism for the induction and maintenance of the inflammatory response associated with disease [2, 3]. For example, deficiencies in efferocytosis have been noted in autoimmune disease such as SLE and COPD [4–15], impaired wound healing in the diabetes mouse model (db) [16] and is associated with chronic inflammation [4, 17]. Efficient efferocytosis is imperative for tolerance induction [18, 19] and defects have been correlated with autoimmunity in mice and humans [2, 4, 20]. Apoptotic clearance is a balance between “eat me” and “don’t eat me” molecular recognition so that live cells avoid being ingested by virtue of molecules that signal macrophages to avoid them such as interaction of CD47 expressed on live cells with Signal Regulatory Protein alpha (SIRPα) on macrophages [21–25]. Additionally, cell type and a regulated balance of positive and negative signals to the macrophages determine whether interaction results in either a pro- or anti-inflammatory response [26].

Macrophage phenotypes and functions are malleable depending on environmental conditions; for example an inflammatory response may be ameliorated by exposure to apoptotic cells [27–34]. This opens the possibility for shifting from an inflammatory to anti-inflammatory response by controlling macrophage phenotypes, for example, by specifically interacting with cell surface molecules involved in apoptotic cell uptake [35]. This is supported by the recent finding that the mechanism of fluticasone, a glucocorticoid used as an anti-inflammatory agent, is through modulation of SIRPα expression and subsequent apoptotic cell uptake [36]. However, adverse effects are common with fluticasone due to the response to steroids in general making it desirable to obtain the same effect in a more specific manner, for example targeting molecules that could potentially enhance efferocytosis [30]. In contrast, it may be advantageous to inhibit apoptotic cell uptake in tumors in an effort to promote an anti-tumor response [37]. Ectopic expression of the TAM receptors, notably MerTK, in tumors confers the ability to engulf apoptotic cells which works in conjunction with macrophages to efficiently remove apoptotic cells. The enhanced kinetics of efferocytosis is one way to potentially promote tumor survival; maintaining an anti-inflammatory environment by down regulating the local immune response [38]. Lack of MerTK, for example using knock-out mice, can result in more effective control of tumors [39]. In the MMTV PyVmT mouse model of breast cancer, an increase in tumor cell death is seen in the absence of MerTK which is most likely due to inefficient efferocytosis as the tumor cells do not express appreciable levels of MerTK themselves. This is supported by data from co-cultures of macrophages and HSV-TK expressing tumor cells treated with ganciclovir to induce apoptosis in which treatment with anti-MerTK resulted in impaired efferocytosis [40].

There is a growing interest in targeting members of the TAM receptor family of receptor tyrosine kinases (**T**yro, **A**xl and **M**er) because they play a role in immune homeostasis, in part through modulation of macrophage function including apoptotic cell uptake [41]. A recent report using mouse models has demonstrated treatment of macrophages with activating antibodies to MerTK alone did not impact apoptotic cell uptake even though the signaling pathway was initiated [42]. Phagocytosis of apoptotic cells required the presence of the soluble adaptor ligand Gas6 in addition to antibody treatment. In a mouse model of collagen-induced arthritis (CIA) increasing levels of the TAM receptor ligands Gas6 or ProS as a means of agonizing the TAM receptors has been shown to decrease the severity of disease [43].

We have developed a system to discover novel molecules that control efferocytosis on macrophages by screening for antibodies in a functional assay that quantitates the phagocytic activity of macrophages. Mice immunized with primary human macrophages derived from healthy donors were used to generate hybridomas; the antibodies were subsequently screened to identify those that impacted the phagocytosis of apoptotic cells by comparing the phagocytic index (PI) of 1° human monocyte-derived macrophages among the entire antibody panel. Incorporation of high content imaging permitted screening of hundreds of antibodies to identify those that either positively or negatively impacted efferocytosis. Such high content imaging-based assays have been described for phagocytosis of bacteria by macrophages, however application to apoptotic cell uptake presents additional challenges that include complications using pHrodo for labeling of apoptotic cells compared with bacteria, and precise timing of the induction of apoptosis to avoid shifting to necrosis during labeling and running the phagocytosis assay. Using this strategy we have discovered two antibodies that enhance efferocytosis and recognize recombinant MerTK. This enhancement of efferocytosis was observed without the addition of exogenous Gas6 or ProS suggesting the potential for antibody-mediated agonistic activity. Since this approach does not depend on prior knowledge of any of the molecules, it can be applied to discovery of novel molecules that regulate this fundamental macrophage function and subsequent impact on the inflammatory response.

## Materials and Methods

### Ethics statement

Animals at Janssen Research & Development, LLC were maintained in a facility approved by the American Association for Accreditation of Laboratory Animal Care in accordance with current regulations and standards of the U.S. Department of Agriculture. The protocol was reviewed and approved by the Janssen Research & Development, LLC. Institutional Animal Care and Use Committee.

### Generation of human macrophages

Macrophages were derived from human monocytes isolated from healthy donors by Biological Specialty Corp. (Colmar, PA USA) and frozen in aliquots of 10^7^ cells/vial. Monocytes were thawed in 37° water bath then placed directly into serum free XVIVO-10 (Lonza, Walkersville, MD USA) and plated into tissue culture flasks at a cell density of 1x10^5^ cells/cm^2^. Cells were incubated at 37°C for 1.5 hours then media was decanted and replaced with XVIVO-10+10% FBS (Heat Inactivated, Certified: Live Technologies, Grand Island, NY USA) supplemented with 100ng/ml M-CSF (Peprotech, Rocky Hill, NJ USA) then placed into the TAP Biosystems CompacT SelecT Automated Cell Culture System (Sartorius Stedim, Hertfordshire, UK). The SelecT was programmed to replace 50% of the media on day 4 with an equal volume of XVIVO-10 + 10% FBS supplemented with 10ng/ml M-CSF. Flasks were collected at day 6 then cells were dissociated using Accutase (Sigma-Aldrich, St. Louis, MO USA) according to product instructions, counted then re-plated into 96-well BD Falcon imaging plates having black wells with clear bottom at 20,000 cells/well in XVIVO-10 + 10% FBS + 10ng/ml M-CSF then cultured overnight at 37°C. For generation of M1-M2 macrophages 100ng/ml IFN-γ (Peprotech, Rocky Hill, NJ USA) was added to the media during re-plating. Preparation of macrophages for immunization of mice was done using the same methods except that cells were kept in flasks and underwent a programmed feeding on day 6 with media containing 100ng/ml IFN-γ then harvested on day 7. For immunization the day 7 macrophages were washed, counted and resuspended in PBS at 10^7^ cells/ml. Mice were immunized with 10^6^ cells s.c. at the base of the tail.

#### Macrophage phenotyping

Monocyte-derived macrophages were phenotyped using flow cytometry on day 7 of culture. Antibodies to cell surface markers were as follows: α-CD64-APC-H7, α-CD14-PC5.5, α-CD11b-v450, α-CD206-APC, α-CD68-FITC, α-CD23-PC7, α-CD45-AF700, α-CD16-FITC and α-CD64-PE and relevant isotype controls for each all from BD Biosciences (San Jose, CA USA); α-MerTK- PE was purchased from R&D Systems (Minneapolis, MN USA). Live/Dead Aqua was purchased from Invitrogen (Grand Island, NY USA).

### Preparation of apoptotic cells

Apoptosis of Jurkat cells was induced by culturing at 1x10^6^ cells/ml for 3 hours at 37°C 5% CO_2_ in RPMI 1640 + 10% HI FBS (Gibco, Grand Island, NY USA) in the presence of 1.0μM staurosporine (Trevigen, Gaithersburg, MD USA) [44]. A duplicate culture was concurrently set up to use as live control cells in the phagocytosis assay. Both apoptotic and live cells were labeled with pHrodo SE (Invitrogen, Grand Island, NY USA) [45] using 0.1μM dye in HBSS containing 1mM HEPES pH 7.4 at 10^8^ cells/ml and incubated for 15 minutes in the dark at room temperature. Cells were centrifuged then resuspended in media for use in phagocytosis assays. Prior to pHrodo SE labeling, an aliquot of both live and apoptotic cultures was reserved for active caspase-3 staining using the FITC-Active Caspase-3 Apoptosis Kit (BD Biosciences, San Diego, CA USA) staining to confirm induction of apoptosis [46]. A second method compatible with imaging to confirm apoptosis by staining for active Caspase-3 was performed using the Magic-Red Caspase 3/7 Detection Kit (ImmunoChemistry Technologies LLC, Bloomington, MN USA)

### Immunizations and hybridoma generation

BALB/c mice were immunized with M1-M2 human intermediate macrophages derived from monocytes. Mice received 4 s.c. immunizations at the base of the tail on Days 0, 167, 203 and 252 with 1x10^6^ cells in 1xPBS in a total volume of 100–200μl. Six days prior to splenic harvest, mice received cells in the same manner mixed with 50μg of agonistic α -CD40 antibody (MAB440, R&D Systems, Minneapolis, MN USA) that has been shown to increase the number of antigen-specific hybridomas [47]. Spleen and lymph nodes from immunized mice were collected on Day 279 and processed to single cell suspension to be used in hybridoma generation.

Hybridoma cells were produced following conventional hybridoma formation methods of De St. Groth [48]. Briefly, red blood cells (RBC) were lysed using RBC lysing buffer (Sigma, St. Louis, MO USA) and isolated lymphocytes were combined with FO murine myeloma cells at a 1:1 ratio for fusion. Cell fusion was performed utilizing PEG 4000 and hybridoma growth performed under HAT selection.

### Sera Titers and specificity ELISAs

Sera from mice immunized with M1-M2 intermediate macrophages were assessed for antibody titers using ELISA specific for the recombinant proteins MFG-E8, LRP-1 cluster II, Stabilin-2, Bai 1and SIRPα. Hybridoma supernatants were tested for binding to MerTK in addition to SIRPα and LRP proteins. All of these recombinant proteins were purchased from R&D Systems (Minneapolis, MN USA). Briefly, 96-well ELISA plates (NUNC cat#446612) were coated with 100μLwell each antigen diluted to 10μμg/mL in NaHCO_3_ coating buffer. They were then incubated at 4°C overnight. The following day plates were blocked using 0.4% BSA/PBS without removing coating solution then incubated at least 1 hour at room temperature. Following the blocking step, plates were washed 1x in wash buffer (0.2% Tween 20/PBS). Serial dilutions of sera from each mouse were added to the plates in duplicate starting at 1:100 dilution with serial 5-fold dilutions down the plate then incubated at room temperature for 2 hours. For antibody testing, neat supernatant from purified antibodies was added to each well. After washing plates 3x in wash buffer, detection reagent (HRP-anti-mouse IgG H+L) diluted 1:10,000 was added then plates were incubated for an additional 2 hours at room temperature followed by 3 washes using wash buffer. The OPD substrate (Sigma Aldrich, St. Louis, MO USA) was then added and plates were allowed to sit at room temperature for approximately 15 minutes. Reaction was stopped using H_2_SO_4_ at a final concentration of 1N then results were read using a Powerwave HT340 (Biotek, Winooski, VT USA) at 490nM and Gen5 software (Biotek, Winooski, VT USA).

### MesoScale Discovery (MSD)-based macrophage binding assay

IFNγ activated monocyte-derived macrophages were seeded in PBS at 2x10^4^ cells per well in a 384-well High Bind Plates L15XB-30/L11XB-3 (MesoScale Discovery, Gaithersburg, MD USA). The seeded plates were incubated at ambient temperature for 2 hours to allow for binding to the carbon electrode surface and then blocked with 10% fetal bovine serum in PBS with 0.09% sodium azide to decrease internalization for 15–30 minutes. Hybridoma supernatant was then carefully added to minimize disruption of the cell layer without washing. The cells and supernatants were incubated for 1 hour to allow for binding and then washed with PBS. Ruthenylated (MSD sulfo tag) detection antibody (1 μg/mL) was added for 1 hour at ambient temperature to detect the binding complex Following a second wash, MSD 1X read buffer (MesoScale Discovery R92TD) was added and the binding signal was acquired on a MesoScale Discovery SECTOR Imager 6000 and data analyzed in Excel and visualized using Third Dimension 3DX software

### Purification of antibodies from hybridoma supernatants

Dynabeads (Protein G DynabeadsTM, Life Technologies, Carlsbad, CA USA) were prepared by washing and resuspending in 2-(N-morpholino)ethanesulfonic acid (MES) (Sigma-Aldrich, St. Louis, MO USA) (0.5 M MES Solution pH 5.0; 0.5% Tween-20). Supernatants were prepared by adding 100 μL of 10X MES to 1 mL of supernatant and 100 μL of prepared beads was added to the mixture. For consistency, samples were mixed using the Biomek FX for 60 min (5 cycles of 20 mixes with a pause of 180 sec between cycles, mixing 200 μL per aspiration). The beads were subsequently processed on a KingFisher96 magnetic bead processor (Thermo Scientific, Waltham, MA USA) for the following steps: 5 min binding, 10 min wash with MES, 10min wash in 0.1 M Sodium Acetate and 15 min elution in 150 μL 0.1 M Glycine HCL pH2.5. Each step incorporated gentle agitation with the magnetic probes moving within the wells to ensure even mixing throughout the incubation steps. The eluates were then purified over ZebaTM (Pierce, Rockford, IL) spin desalting plates using the manufacturer recommended protocol and BSA was added to a final concentration of 0.5% in the final eluates.

### High content phagocytosis assay

Monocyte-derived macrophages were seeded in media at 2x10^4^ cells per well in 384-well imaging plates (Perkin Elmer, Waltham, MA USA). For the positive control, dexamethasone (10μM) was added to cells and incubated at 37°C 5% CO_2_ for 16–20 hours prior to assay set up. When testing the panel of antibodies, purified IgG from hybridoma supernatants was added to macrophages at 5μL/well followed by a 2 hour incubation at 37°C 5% CO_2_ prior to addition of apoptotic cells. Apoptotic Jurkat T cells were added to the wells containing macrophages and antibodies at a ratio of 5:1 (Jurkat T cells to macrophages) and co-cultured for 30 min at 37°C in 5% CO_2_. Negative control wells were treated with Cytochalasin D (2μM) [29]. Following the 30 minute incubation all wells were washed with cold PBS, then stained with an antibody cocktail consisting of CD11b-APC, CD14-APC and CD64-APC followed by counterstaining with Hoechst. Representative fields per well were imaged using a Perkin Elmer Operetta High Content Imager and with quantitation using Perkin Elmer Harmony or Columbus analysis software. On average, 200 macrophages were analyzed per duplicate for each sample. A phagocytic index (PI) was calculated by multiplying the percentage of macrophages that phagocytosed a target by the average number of targets engulfed per macrophage [49].

$$\frac{100\;x\;\#\;macrophages\;with\;engulfed\;targets}{Total\;\#\;macrophage\;}x\frac{Total\;\#\;engulfed\;targets}{Total\;\#\;macrophage}$$

In assays testing effect of Gas6, MerTK and MFG-E8, recombinant proteins were purchased from R&D Systems (Minneapolis, MN USA) and added to the macrophages prior to addition of apoptotic cells. Pre-treatment with MFG-E8, Gas6 or Gas6 + MerTK-Fc was for 20 minutes then apoptotic cells were added and incubated for an additional 20 minutes after which plates were washed and analyzed as described above. In parallel, macrophages were pre-treated with a human IgG control, CNTO 360 which was generated in house.

Gating strategy (S1 Fig) was as follows: Find macrophages based on APC stain; Define cell morphology; Select this population (Cells Selected); Define cell region (exclude outer 5% from analysis to minimize counting Jurkat cells not fully engulfed; Find Jurkat cells based on pHrodo stain; Define this population by size (Ingested Cells); Define properties of Ingested Cells based on size and fluorescence intensity (Spots); Properties of Cells Selected by related population (Spots); Count macrophages as positive if they contain ≥ 1 spot/cell.

### Statistical analysis

Statistical analysis included one way ANOVA, Dunnett’s multiple comparisons test and Tukey’s multiple comparisons test all performed in GraphPad Prism 6.

## Results

### High content imaging as a quantitative means for measuring efferocytosis

**Fig 1:**
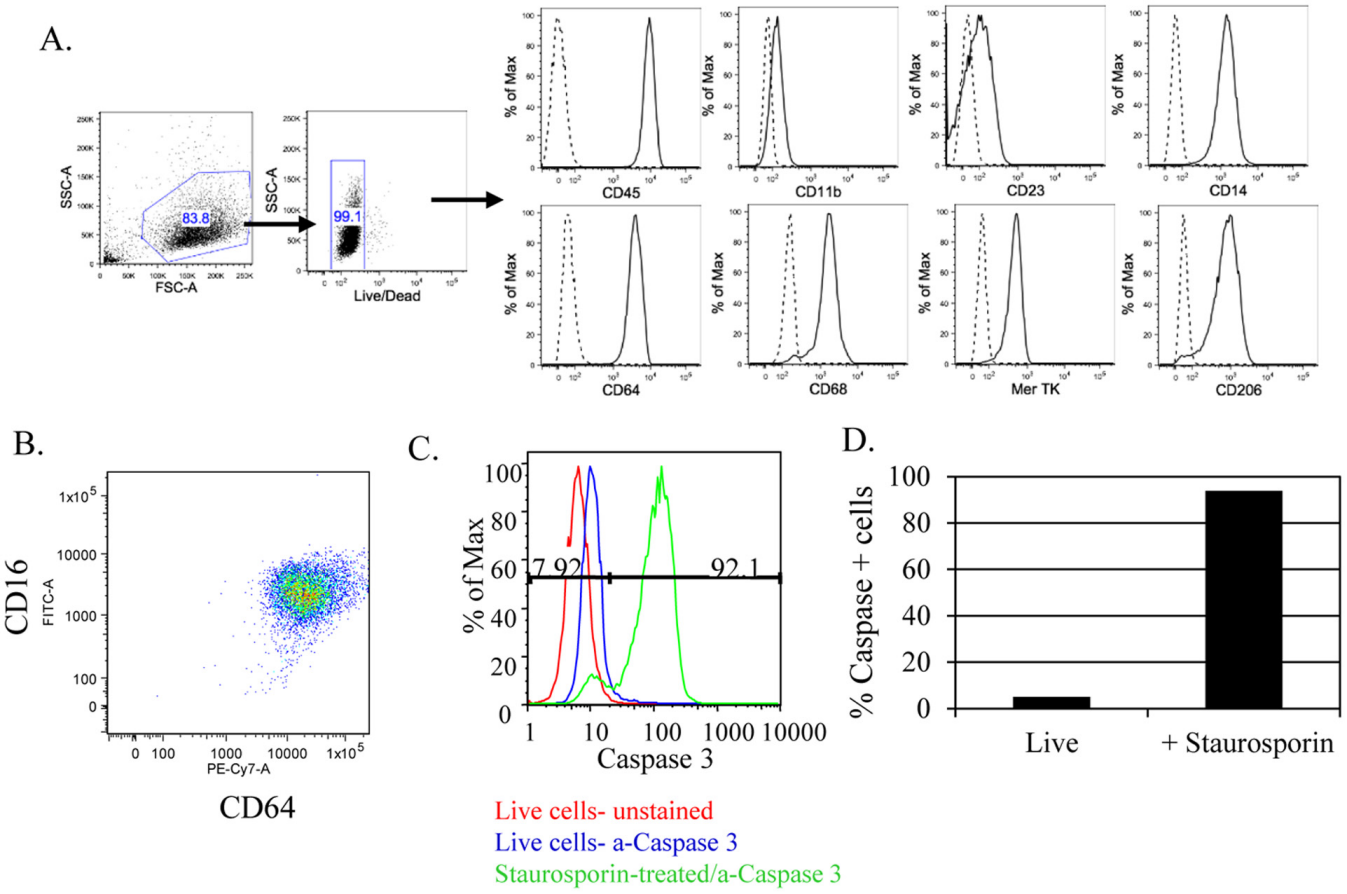
Characteristics of SelecT generated macrophages and apoptotic status of staurosporine-treated cells. Macrophages were prepared by culture with M-CSF as described in materials and methods. Following treatment with IFN-γ, the majority of macrophages express markers found on both M1 and M2 populations (A). Cells were gated by scatter then expression of markers was assessed on live cells. M1-M2 intermediate phenotype macrophages are CD64^+^CD16^+^ (B). Jurkat cells were treated with staurosporine for 3 hours at 37°C as described in materials and methods. Cells were stained for active caspase 3 then quantitated using flow cytometry (C) or stained with Magic Red then measured using high content imaging (D).

Assessment of apoptotic cell uptake competence in macrophages as a means of testing antibody panels requires a robust and reproducible method to induce apoptosis and a process for generating macrophages in quantities that can accommodate screening panels of antibodies consisting of hundreds of molecules. Macrophage-like cell lines are available; however, they do not consistently retain all of the functional traits of 1° macrophages [50–54]. Monocyte-derived macrophages were cultured in the SelecT automated cell culture system using M-CSF, and then treated with IFN-γ for the final 24 hours for a total culture time of 7 days. As shown in Fig 1 the majority of cells differentiated to macrophages that express markers associated with both M1 and M2 populations [55, 56]. In particular, the macrophages co-express CD64 and CD16 which is characteristic of an M1-M2 intermediate phenotype.

To focus on identification of antibodies that specifically impact phagocytosis of apoptotic cells, the conditions to induce apoptosis were carefully controlled as macrophage recognition and uptake of necrotic cells differs from that of apoptotic cells [57, 58]. As an early event in apoptosis, caspase-3 activation was used as the key indicator during optimization to monitor induction of apoptotic conditions [44]. This was confirmed using two different methods for detection of active caspase 3; the first method used flow cytometry and staining cells with an anti-caspase mAb that recognizes caspase-3 specifically. The second method is based on cleavage of a caspase-3/caspase-7 substrate peptide (DEVD) consisting of two repeats in tandem and engineered so that a fluorescent signal (Magic Red^™^) is emitted upon cleavage. Previous reports show that apoptosis induced by staurosporine is detectable at 3 hours [44] using 2.5 μM solution. To determine the optimal conditions for apoptosis induction using staurosporine that would induce apoptosis without driving the cells beyond early apoptosis, a dose-response study was performed using lower staurosporine concentrations and treating for 18 hours; however, this resulted in cell death (S2 Fig). It is important to use the cells prior to progression to a late-apoptotic state because late-stage apoptotic cells have an increased chance of progression to death during the course of the assay which could impact the results. As shown in Fig 1c and 1d, optimal conditions identified for induction of apoptosis by treatment with staurosporine yielded ≥90% apoptotic cells as defined by caspase activation using either detection method. Note that both caspase-3 detection methods were tested in the same experiment to confirm the Magic Red technology and antibody-based method gave comparable results for quantitating the percentage of apoptotic cells. Having two methods for reliably detecting apoptosis gives the option of checking the apoptotic condition of the input cells by flow cytometry or concurrent with imaging analysis of uptake by macrophages.

**Fig 2:**
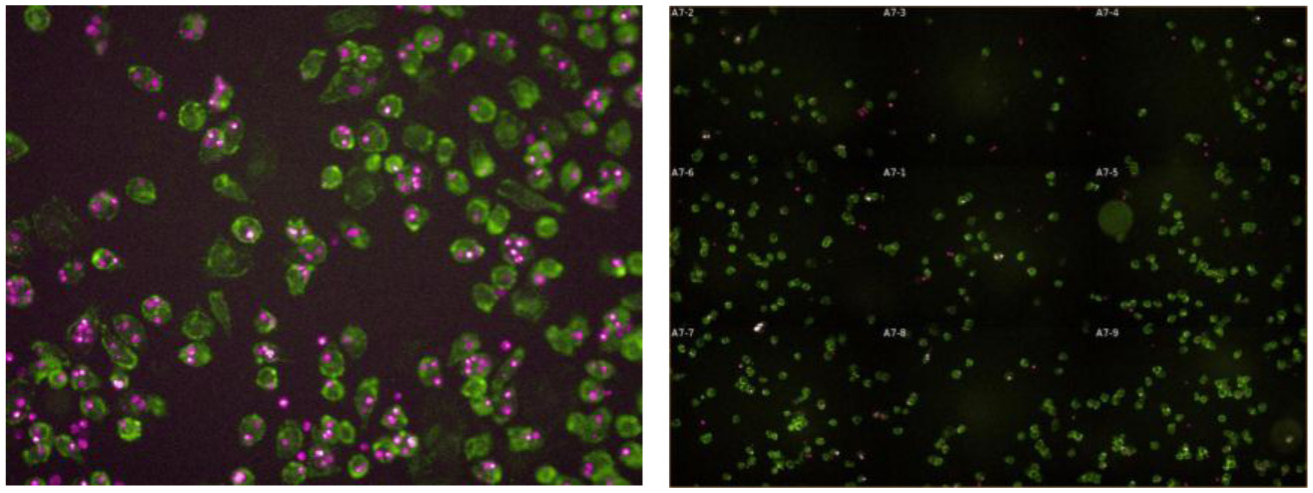
Intact apoptotic cells are detectable within macrophages. Macrophages were co-cultured with apoptotic Jurkat cells labeled with pHrodo dye for 30 minutes. Excess Jurkat cells were washed away then macrophages were fixed and stained with a cocktail of antibodies consisting of anti-CD14-APC, anti-CD64-APC and anti-CD11b-APC. Macrophages (APC^+^) appear green in the image and apoptotic Jurkat cells (Cy3^+^) appear red. On the left is a representative image from a single field of 6 total image fields taken per well of a 96-well plate (40X objective) and has been enlarged to show macrophages that have engulfed apoptotic Jurkat cells. Image on the right showing macrophages co-cultured with live Jurkat cells includes all 6 fields of a well to demonstrate the paucity of engulfed cells. Images were captured using the Operetta High Content Imager. Confirmation of active phagocytosis during the 30 minute incubation is demonstrated in S1 Movie.

Establishing a quantitative assay to measure the effects of antibodies on apoptotic cell uptake by macrophages requires capturing images early after engulfment to avoid spurious data due to digestion of the engulfed cells. Target cells, both apoptotic and live, were labeled using pHrodo [45] to facilitate visualization then incubated with macrophages. To identify conditions of maximum engulfment but minimum cellular degradation a time course was initially performed over a period of 40 minutes and samples were collected for analysis at 10 minute intervals (S3 Fig). At each time point, cells were washed with cold PBS containing sodium azide to facilitate removal of any apoptotic cells adhering to the surface of the macrophages and impede breakdown of internalized material. Samples were subsequently fixed and stained with the CD11b/CD14/CD64 antibody cocktail to identify macrophages and DAPI to enumerate nuclei as described in materials and methods and (S1 and S3 Figs). The images shown in Fig 2 verify that intact cells are visibly contained within the macrophages after 20–30 minutes of co-culture. Live cell imaging in real time confirms macrophages are engulfing the apoptotic cells (S1 Movie).

**Fig 3:**
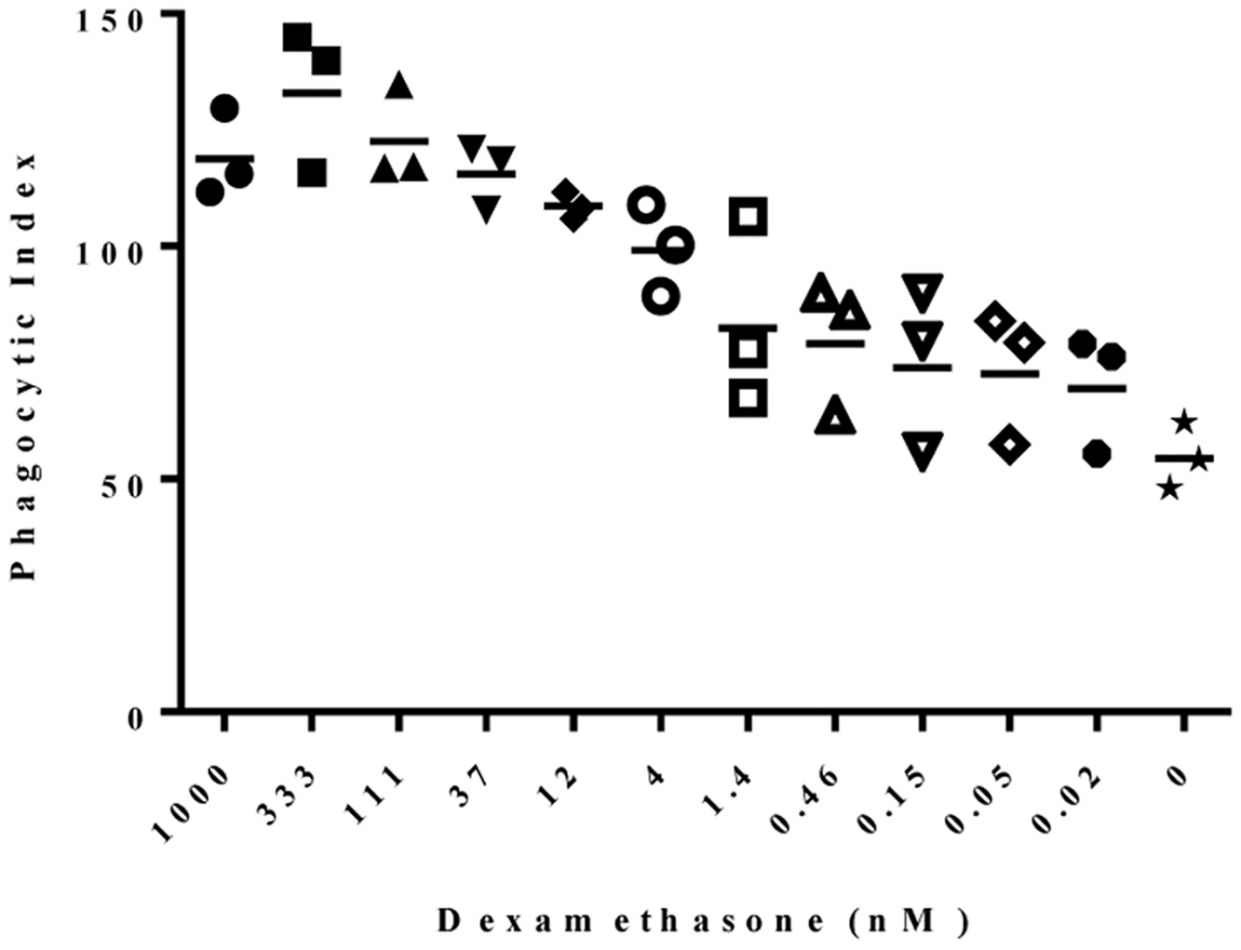
Dexamethasone treatment impacts the ability of macrophages to engulf apoptotic cells in a dose-dependent manner. Macrophages were treated with varying doses of dexamethasone then imaged following 30 minute incubation with apoptotic Jurkat cells as described for Fig 2. Phagocytic Index (PI) was calculated at each dose from 1 field/well collected using the Operetta High Content Imager. PI = (Total # engulfed cells/Total # counted MΦ) * (# MΦ’s containing engulfed cells/Total # counted MΦ) * 100 [49].

To determine if a dose-response in the PI could be quantitated using high content imaging, macrophages were treated with increasing concentrations of dexamethasone which has been reported to enhance phagocytic activity [59]. Dexamethasone treatment was performed with a starting concentration of 1μM and serial diluted down to 20pM then the treated macrophages were assessed for relative capacity to engulf apoptotic Jurkat cells. Calculation of the PI indicates that a dose-response was evident in the concentration range of 111 nM-1.4 pM, (Fig 3). Treatment with dexamethasone at concentrations >111nM resulted in a plateau of the PI. Macrophages treated with dexamethasone concentrations in this range exhibited cell death which most likely accounts for the plateau in PI. Dexamethasone at concentrations below 1.4pM had little impact on phagocytic activity when compared to the PI of macrophages with no dexamethasone.

**Fig 4:**
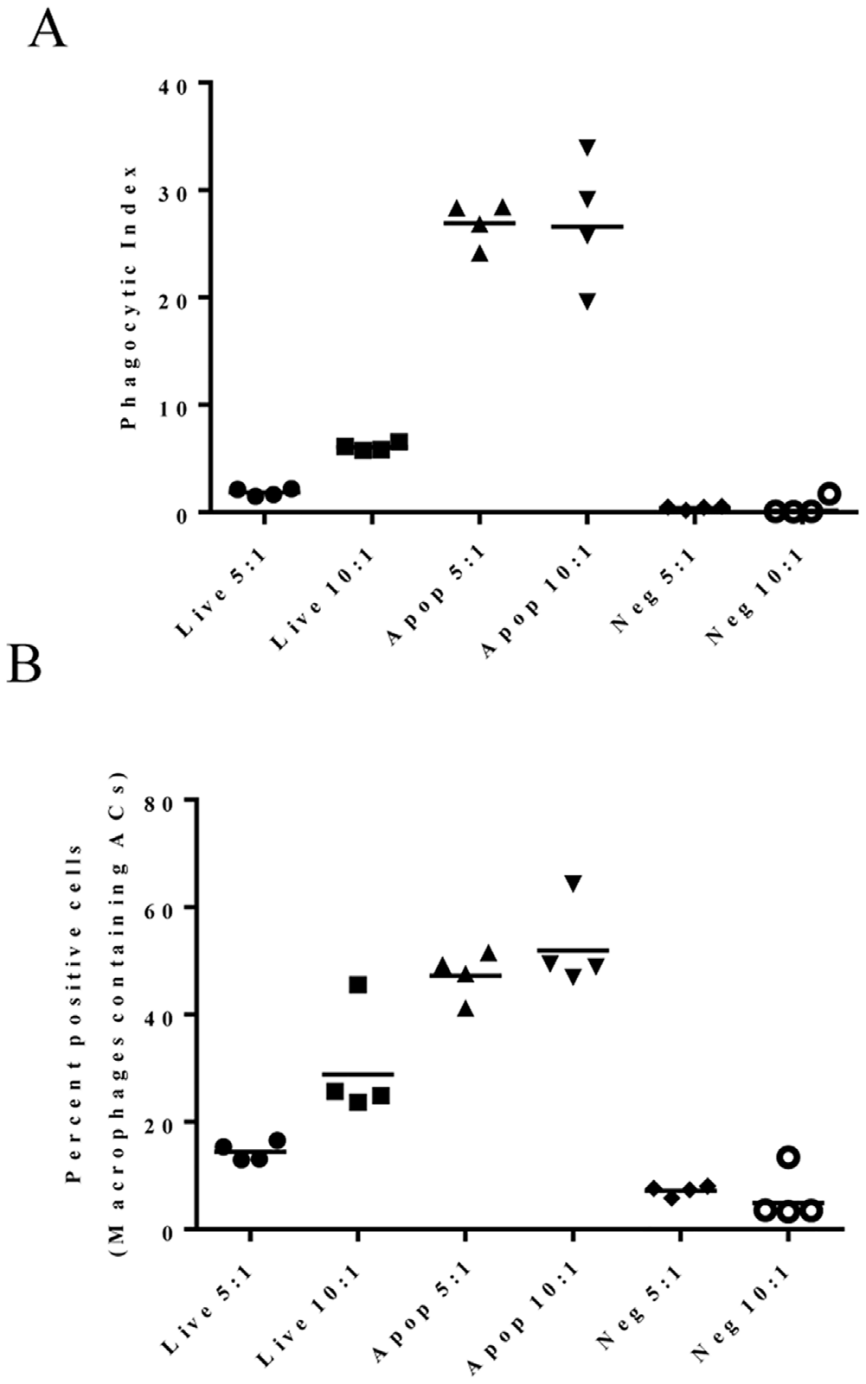
Phagocytic Index (PI) provides a direct measure of macrophage function. Comparison of data presented as either PI (A) or representing the frequency of macrophages containing apoptotic cells expressed as “percent (%) positive cells” (B) within the same population in each well of a 96 well plate. The PI was calculated as described in Fig 3. Calculation of the % positive cells is based on the assumption that macrophages (APC^+^) containing apoptotic cells (Cy3^+^) appear fluorescent for both fluorophores and calculated as follows: % positive cells = (# APC^+^ Cy3^+^ cells / # APC^+^ cells) * 100. Ratios listed along the X-axis represent the (Target) T: MΦ ratios. Baseline engulfment was determined using MΦ’s co-cultured with live cells (Live); Negative controls consisted of cytochalasin D treated MΦ’s co-cultured with apoptotic cells (Neg). Test samples consisted of apoptotic cells co-cultured with MΦ’s (Apop).

Traditionally the PI is considered to be a quantitative measure of phagocytic activity; however, this requires manually counting macrophages that contain engulfed cells on slides using a microscope [60]. To increase throughput, flow cytometry- based phagocytosis assays have been developed which are based on the percentage of events that emit signals from two different labels; one for macrophages and one for apoptotic cells. These data are used to determine the frequency of macrophages that have engulfed apoptotic cells [45, 61, 62]. However, this format is not amenable to quantitating the number of apoptotic cells engulfed per macrophage because the readout consists of only the total signal from apoptotic cells per macrophage with no way of knowing the corresponding number of engulfed cells. To determine whether there is any advantage to looking at the PI rather than total fluorescence of labeled apoptotic cells, the macrophages were analyzed after incubation with pHrodo labeled apoptotic Jurkat cells for 30 minutes. In the same study, macrophages were incubated with pHrodo labeled live Jurkat cells (no staurosporine) to assess baseline apoptotic cell uptake. Due to constant cell death even in healthy cultures, we have observed a minimum of 10–15% apoptotic cells is normally present so incubation with live cells gives an indication of the basal apoptotic cell uptake activity of the macrophages. As a negative control, phagocytosis was blocked in macrophages by treating with cytochalasin D, which inhibits actin polymerization, prior to incubation with pHrodo-labeled, apoptotic, Jurkat cells [29]. The frequency of positive cells (% positive) was determined by reading the total fluorescence/well then compared with the PI calculation obtained for the same well using the Operetta High Content Imager (Fig 4). Estimation of phagocytic activity using total fluorescent measurements can differentiate phagocytic activity in samples having either live or apoptotic cells (compare 5:1 live to 5:1 apoptotic in Fig 4a). This is also true for macrophages incubated with either live or apoptotic cells at 10-fold excess; however the baseline as shown by the macrophages receiving live cells begins to increase. The differences were much more pronounced when cultures were evaluated based on PI (compare 5:1 live to 5:1 apoptotic in Fig 4b) and could be further amplified when macrophages were incubated with 10-fold excess of apoptotic cells. Note that neither method could distinguish between cultures with 5-fold vs 10-fold excess of apoptotic cells at the time point used here. This is most likely due to the rapidity of engulfment in the confines of the well.

### Mice immunized with IFN-γ stimulated macrophages produce antibodies that recognize molecules involved in apoptotic cell uptake

**Fig 5:**
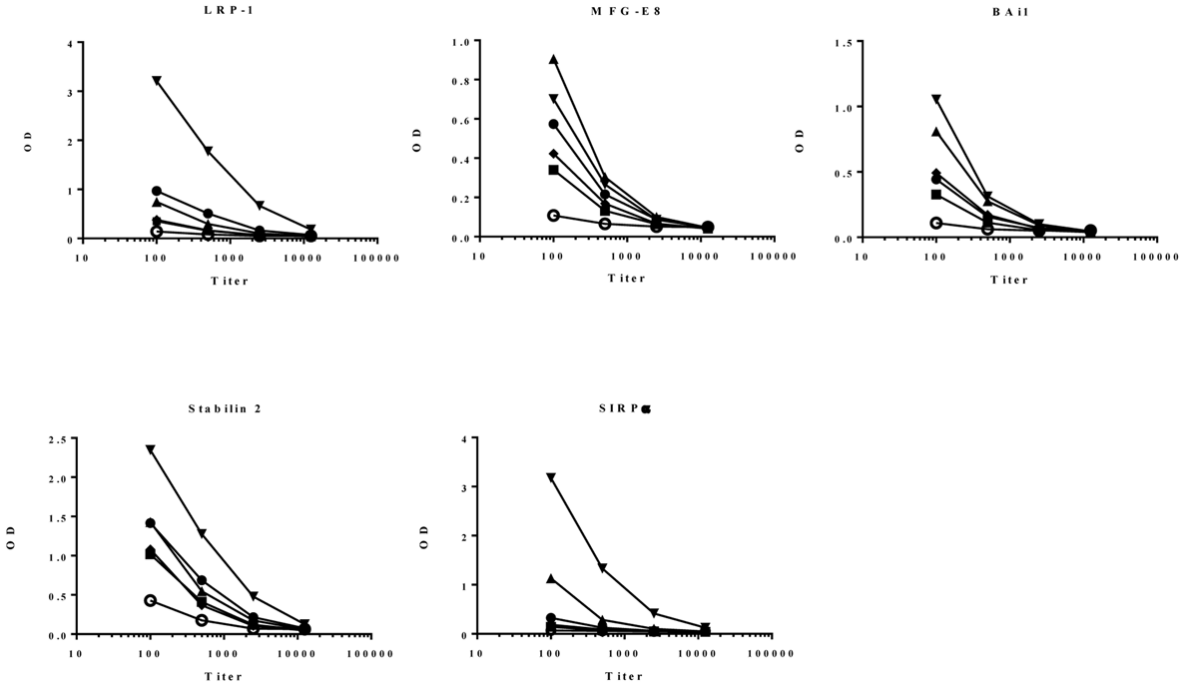
Mice immunized with human macrophages produce antibodies that recognize molecules involved in efferocytosis. Sera collected from mice immunized with human M1-M2 MΦ’s treated with IFN-γ were tested for titers to LRP-1, Bai1, Stabilin 2, MFG-E8 and SIRPα by ELISA. Data are shown for 5 immunized mice with each mouse represented by a filled symbol, and compared to sera from an unimmunized (naïve) mouse represented by the open circles.

**Fig 6:**
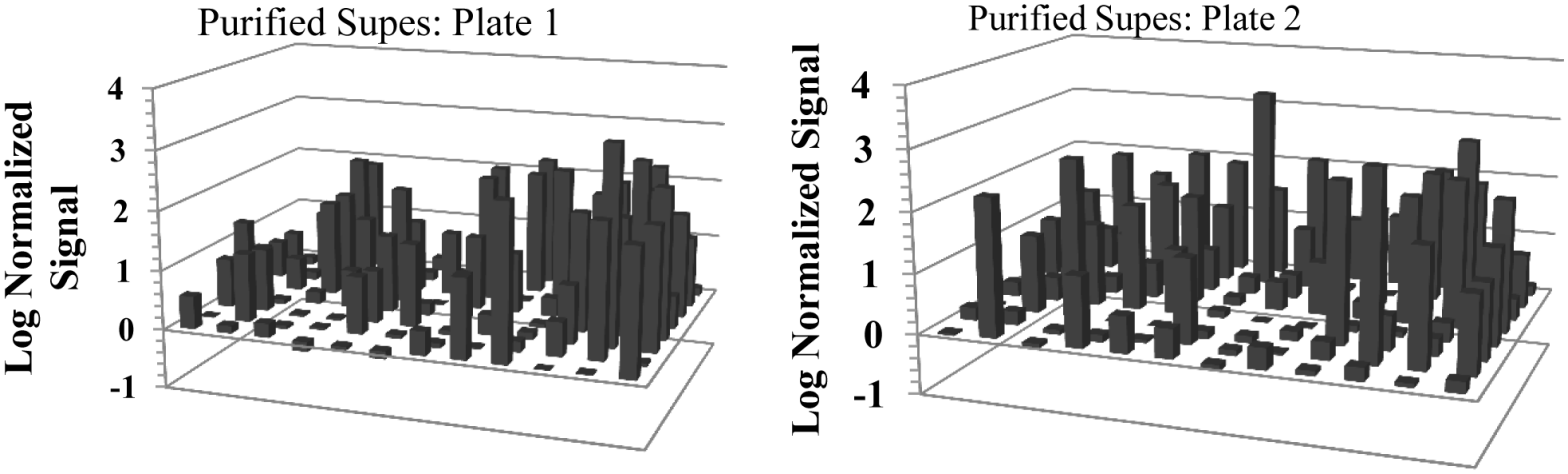
Approximately half of the hybridomas retain MΦ binding following expansion and purification. Supernatants from hybridomas that were expanded and purified were screened on IFN-γ treated MΦ’s using MSD as the readout. All 700 hybridomas were tested, shown above is representative data from two 96-well plates. Histograms are laid out in a 96-well plate format such that the X-axis represents the column numbers and the Z-axis represents the row identities. Each sample was run as a single replicate and log transformed signal ≥ 0.5 was considered positive.

A feasibility study was conducted to determine whether hybridomas generated from mice immunized with human macrophages could be screened based on impact on macrophage phagocytosis of apoptotic cells using this system. Macrophages were generated using the CompacT SelecT and differentiated to M1-M2 intermediate phenotype with IFN-γ prior to immunization. Mice were boosted twice after the primary immunization using preparations of macrophages derived from different donors to favor a response recognizing molecules common to IFN-γ activated macrophages, some of which are potentially involved in apoptotic cell uptake. Measurable titers were detected in response to BAi1, Stabilin-2 and MFG-E8 in all mice (Fig 5) after 3 immunizations [63–65]. One mouse demonstrated titers to all proteins tested and had the best response to both LRP-1 and SIRPα; together mouse 3 and mouse 4 had the highest titers measured to all 5 proteins. As such these two mice were chosen for hybridoma production. Approximately 700 hybridomas were identified as macrophage-specific using a cell-based MSD binding assay. Because of the potential for effects from the hybridoma culture media on macrophage function, those cultures producing macrophage-specific antibodies were expanded for small scale antibody purification then all hits were re-tested so that only those with confirmed macrophage binding were further processed. As shown in Fig 6 approximately 50% of the cultures retained macrophage binding following expansion and purification. The confirmed macrophage binding antibodies were further tested for ability to impact macrophage engulfment of apoptotic cells.

### Utilizing high content imaging to screen antibodies for activity impacting macrophage engulfment of apoptotic cells allows for differentiation based on function

**Fig 7:**
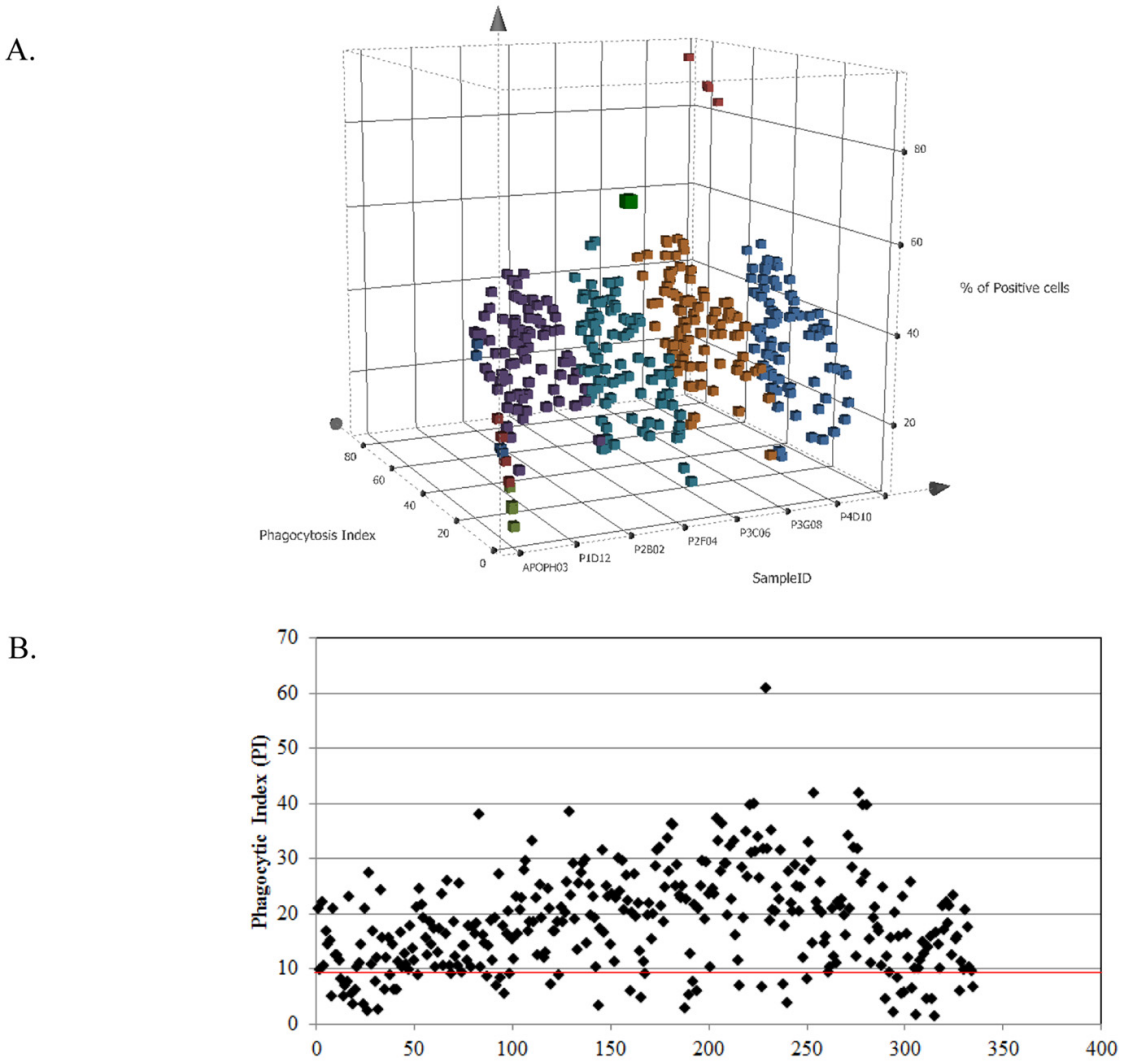
Monoclonal antibodies capable of modulating efferocytosis are identified using high content image-based screening. Purified hybridoma supernatants were tested in a 384-well format using day 7 MΦ’s co-cultured with apoptotic Jurkat cells then analyzed on the Operetta High Content Imager. Results are expressed as PI (z-axis) and % positive cells (y-axis) for each sample (A) with controls represented as follows: cytochalasin D treated MΦ’s with apoptotic cells negative control (green squares); live cell, baseline phagocytosis (dark red squares); apoptotic cells with no antibody (blue squares); dexamethasone treated MΦ’s with apoptotic cells positive control (orange squares). Samples from each plate are grouped and represented by colored squares. The antibody panel contains molecules that exhibit positive and negative effects on MΦ efferocytosis as revealed by comparison of PI of treated cells to controls (B). Each diamond represents the PI data from an individual hybridoma and each is compared to the PI of MΦ’s incubated with apoptotic cells in absence of antibody (red line) which is geometric mean of 4 replicates 9.995 ± 7.54. Data shown are from testing antibodies on macrophages from a single donor, a second assay using cells from a different donor was also performed yielding similar results.

**Fig 8:**
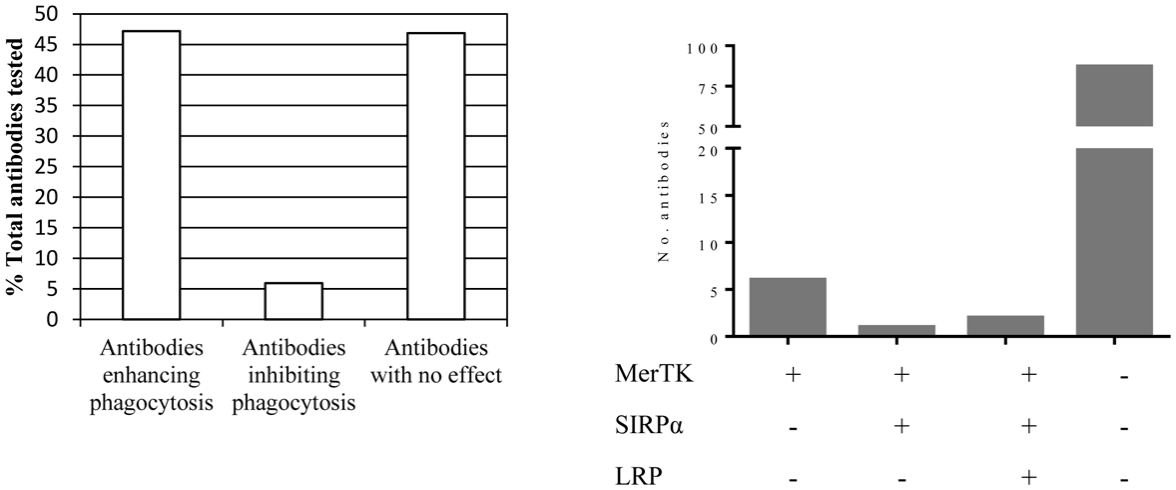
The majority of antibodies enhance apoptotic cell phagocytosis through recognition of unidentified surface molecules. Antibodies having a PI ≥ average PI + 1 SD (standard deviations) for cultures with no antibody treatment are considered as enhancing. Those having a PI≤ average PI—1 SD are considered inhibitory. All other antibodies having PI within 1 SD of average calculated for untreated MΦ’s are considered as having no effect. Frequency of each category of antibody is calculated as percent of total number of antibodies in the panel (left). Binding to known molecules could be detected in a small number of clones (right). Antibodies were evaluated by ELISA and readings that were ≥ 5-fold over background were considered positive.

Using the conditions and set up described above, the entire confirmed macrophage binding panel was tested for ability to either enhance or inhibit apoptotic cell engulfment by macrophages. As shown in Fig 7, the antibodies exhibit unique PI profiles enabling identification of those with desired properties. Within this panel there was a small number that appear to be blocking phagocytosis as the calculated PI fell within the same range as macrophages treated with cytochalasin D (Fig 7a). Most antibodies fell in the middle range in that they did have a measurable impact on enhancement of phagocytosis whether measured as a PI or percent positive cells but many of these differences are unlikely to be statistically significant. The range of activity is more evident when looking exclusively at the PI (Fig 7b). Overall there were 158 antibodies that enhanced phagocytosis and 20 that inhibited phagocytosis. As can be seen by the distribution, the majority of the antibodies functioned to increase apoptotic cell uptake although to varying degrees (Fig 8).

Given that antibody titers specific for several molecules known to be involved in efferocytosis were measurable in the mice used to generate the hybridomas the next question was whether any of these specificities were represented in the panel. To explore this possibility a subset of antibodies was randomly chosen and tested for specificity to LRP-1 and SIRPα as titers to each of these proteins was detectable. Binding to MerTK was also assessed as an additional relevant molecule for which antibody titers were not assessed. The results of these ELISAs are summarized in Fig 8 and demonstrate that only a small number of antibodies recognized these three molecules. It should be noted that all 6 of those that bound only a single molecule were specific for MerTK. Only 2 of the MerTK-specific antibodies enhanced efferocytosis as defined by a PI ≥ Avg+1SD, the remaining 4 antibodies had no measurable effect. We could demonstrate that addition of Gas6 enhanced efferocytosis and this enhancement could be ablated by addition of MerTK-Fc thus confirming that MerTK-mediated efferocytosis can be evaluated using this assay S4 Fig). It is likely that the few antibodies that bound 2 or all 3 of the molecules represent mixed hybridomas as these were not subcloned prior to analysis. Not surprisingly, the specificity of the majority of antibodies could not be determined in this assay.

## Discussion

Herein we present a novel system for discovering molecules that regulate efferocytosis by assessing panels of antibodies for impact on macrophage function. Two antibodies were discovered that modulate apoptotic cell engulfment potentially through interaction with MerTK on the macrophages as they bind to recombinant protein by ELISA. No exogenous ligands were added suggesting that the antibodies are acting through interaction with MerTK itself although thorough characterization is required to confirm this hypothesis. It is possible that MerTK ligands or other factors were present in the media as we used media supplemented with FBS; however, in our experience there is no difference in efferocytosis due to presence of FBS. Our strategy incorporates a quantitative assay based on the Phagocytic Index (PI) which is the parameter traditionally used to measure apoptotic cell uptake. Lack of practicality for assessing large sample numbers has led to alternative methods to accommodate looking at larger numbers of test molecules which enable estimation of macrophage function indirectly, such as use of flow cytometry. Using cell culture automation coupled with development of an image-based assay we have overcome the major obstacle(s) for using PI as a read out for screening. Automated cell culture systems, like the CompacT SelecT, support generation of human macrophages in quantities sufficient for screening antibody panels. This system allowed generation of enough macrophages to run 2–4 96-well plate assays for every 4 T225 flasks of monocytes prepared from healthy donors allowing for high throughput screening of 30 plates. Application of these methods to screening hybridomas enabled identification of antibodies that alter macrophage function, i.e. either enhanced or hampered phagocytosis of apoptotic cells.

Flow cytometry has been successfully employed to assess macrophage function; however, there are several characteristics that make it less than optimal for screening antibodies. Generally this method relies on identification of cells co-stained with macrophage markers and apoptotic cells labeled with fluorescent dye. The dynamic range of this type of assay is limited because the maximum is set at 100% and the minimum at 0% which makes it challenging to differentiate functional impact when assessing a panel. As a result the window is smaller when looking at the percentage of positive cells rather than the PI (see Fig 4). Comparing the PI obtained for live versus apoptotic cells incubated with macrophages at 5:1 target:macrophage ratio results in a difference of ~20-fold whereas the difference for percent positive is ~3.5-fold. Similarly the PI increases ~5-fold whereas the difference seen when calculating the percent positive increases only ~2-fold at the 10:1 target:macrophage ratio. The geometric mean of the PI calculated for cultures having 5:1 compared to 10:1 target:macrophage ratio is indistinguishable in the experiment shown here although the range of PI in the 10:1 ratio sample is much greater than what is calculated for the 5:1 ratio (Fig 4). One caveat for using imaging to calculate PI is that timing is important as engulfed cells are broken down quickly and can appear as multiple spots which will be counted as multiple cells. This can lead to inaccuracies in PI calculation resulting in artificially elevated values. To avoid the potential for spurious inflation in PI calculation, careful consideration must be given to timing of the studies as well as establishing the algorithm for calculating PI. For example when defining the spot size, include only spots representing cells and disregard smaller spots. In establishing our algorithm, we also excluded any cells attached to the outer membrane from the analysis to avoid counting them as engulfed. There is always a minimal level of phagocytosis of untreated cells (live) by macrophages (Figs 2 and 4) which is most likely the result of spontaneous apoptosis due to normal cell turnover. Incorporating this control provides an indication of the overall health of the culture of target cells.

It should be noted that the time of incubation will vary depending on cell type and possibly also donor. Many protocols use a two hour incubation to assess PI but the readout is via manual assessment using microscopy. This allows differentiation of larger, whole cells from smaller fragments due to breakdown of engulfed cells. However this distinction/capability is not intrinsic to algorithms used to analyze images. It can be built into the algorithm to eliminate the cell fragments that appear as small spots so that the overall PI is not artificially elevated. As an additional precaution we kept the incubation time down to 20–30 minutes of co-culture and washed with cold PBS containing azide to minimize breakdown of the engulfed cells between the time of the culture and analysis.

### High Content Image-based calculation of PI can be used to select for antibodies that can impact macrophage function

Phagocytosis of apoptotic cells was used as a functional readout to screen for antibodies that modulate macrophage function with no knowledge of binding specificity. The ultimate goal is to apply this assay to identify previously unknown molecules involved in apoptotic cell uptake that could potentially serve as novel therapeutic targets. As a pilot study for finding macrophage-associated targets mice were immunized with macrophages derived from normal donors. To increase chances of finding antibodies targeting molecules mediating apoptotic cell uptake treatment with IFN-γ was performed to polarize the macrophages to M1-M2 intermediate since both populations can engulf apoptotic cells to some degree [66]. We could detect antibody titers in mice immunized with macrophages directed at a subset of molecules known to be involved in apoptotic cell phagocytosis. This provided confidence that antibodies could be obtained based on ability to modulate phagocytic activity of macrophages.

Binding to macrophages was confirmed prior to the functional testing to ensure only macrophage-specific antibodies were further analyzed. This is an imperative step as the hybridoma supernatants cannot be directly incorporated into the functional assay due to presence of many unknown factors in the hybridoma media. Presence of this media could impact the viability and function of the macrophages independent of the antibodies. Therefore the antibodies require a scale up step from a 96-well culture in order to generate enough material for small scale purification and scaling up all 2640 hybridomas to screen in the functional assay was impractical. Eliminating the non-binders up front reduced the number of cultures to be scaled up to approximately 700 which corresponds to approximately 35% of the starting number. Following scale up and purification the purified antibodies were retested to confirm macrophage binding. The reason for this is that the hybridomas were not subcloned to select for single clones meaning it is highly likely that a fraction of the wells contained more than one clone and a mixture of antibodies. Efforts were made to eliminate any clones that either did not produce antibody or produced antibody that lacked macrophage binding activity in the primary assay but outgrew the other(s) in the well. We postulate that the outgrowth of such clones is most likely the reason that only approximately 50% of the cultures retained macrophage binding after scale up.

Analysis of the set of antibodies based on PI shows that the majority were able to enhance apoptotic cell phagocytosis; only a small subset of antibodies could be categorized as potential inhibitors of phagocytosis (Fig 8). The preponderance of efferocytosis-enhancing antibodies is perhaps owed to the fact that (1) this strategy is focused on macrophage function and (2) the antibodies are being generated using native proteins in their naturally occurring context. We are not aware of any studies that take this approach of subjecting hundreds of antibodies to a functional screen for impacts on efferocytosis although smaller studies have been reported [67]. This type of approach would normally be cumbersome but the adaptation of automated cell culture technology permits generation of macrophages in sufficient quantities for both immunizations and screening in phagocytosis assays using only primary cells. Additionally the use of 1° macrophages for immunization and screening provides the advantage of working exclusively with native rather than recombinant proteins. Perhaps there is a subtle difference between some of the native and recombinant proteins and/or the context in which they appear on the macrophages that provides an advantage. That many antibodies had no effect on apoptotic cell uptake by macrophages is not surprising since the hybridomas were generated from mice immunized with bulk macrophages expressing varying levels of relevant molecules acting as antigens. It is interesting that of the antibodies that impacted the PI, the vast majority enhanced apoptotic cell phagocytosis, outnumbering those that inhibited the PI by almost 8-fold. Of the 20 clones that inhibited phagocytosis, only 2 had a PI equivalent to that obtained for the cytochalasin D treated negative control. The reason for this is unclear; however it will be interesting to generate additional antibody panels to determine whether this is a meaningful trend or unique to the conditions of this study.

Although the possibility that the MerTK-specific antibodies enhanced efferocytosis through direct binding to the Jurkat cells cannot be ruled out, we think this is unlikely. One scenario is that the antibodies bind to MerTK on Jurkat cells [68] and enhance phagocytosis through FcR interaction on the macrophages. The antibodies tested here are mouse IgG and in the MSD binding experiments binding to human FcR’s on macrophages is at the threshold of detection although this does not completely eliminate the possibility that some of the antibodies in the panel are functioning through an FcR-mediated pathway. An alternative scenario could be that the MerTK specific antibodies enhance efferocytosis indirectly by augmenting apoptosis of the Jurkat cells. Since the majority of Jurkat cells (90%) were apoptotic at the time of the assay any increase through MerTK interaction would be nominal and unlikely to noticeably impact efferocytosis.

It is possible that some of the antibodies in the panel are functioning through FcR-mediated interaction. This can be envisioned via two potential scenarios; (1) The Fc of the antibodies could interact with the FcR on the macrophage as noted previously or (2) anti-FcR-specific antibodies acting in an agonistic manner. It is likely that the panel generated by immunizing mice with macrophages yielded some frequency of antibodies specific for FcRs. A subset of those antibodies could potentially be agonistic, particularly if the Jurkat cells express FcR. As no assessment of FcR expression was performed on the Jurkat cells we cannot entirely rule out this possibility. Regardless, it is most likely that several different MOAs for impacting efferocytosis are represented by this panel of antibodies.

In order to harness the potential to treat inflammatory conditions by enhancing the ability of macrophages to clear apoptotic cells, much still remains to be learned. It is conceivable that only a fraction of the molecules on macrophages that are involved in efferocytosis have been identified making the approach described herein vital to discovery of others [69]. Though the binding specificities of most of these antibodies are unknown, a few antibodies were found to bind to MerTK by ELISA. Although further characterization is required to determine whether there is any impact on macrophage phenotype as it pertains to inflammation, This result is encouraging since MerTK is known to be involved in apoptotic cell clearance [70, 71]. There is evidence that the functional phenotype of macrophages is not permanent which, together with these data, opens the possibility of using antibodies to ameliorate an inflammatory response through alteration of macrophage function such as efferocytosis [72, 73].

## Supporting Information

S1 Fig Segmentation and gating strategy for obtaining PI based on quantitating engulfed cells. Analysis was performed using Columbus software and sample gates shown are as follows: (A) Find macrophages based on APC stain to define and select this population. (B, C) Define Cell region to minimize counting Jurkats not fully engulfed, find Jurkats based on pHrodo stain and define this population by size (Ingested Cells) and fluorescence intensity (Spots) and select this population (Spots). (D) Count macrophages as positive if they contain ≥ 1 spot/cell. (E) Intact apoptotic cells are detectable within macrophages. Macrophages were co-cultured with apoptotic and live Jurkat cells labeled with pHrodo dye for 30 minutes. Excess Jurkat cells were washed away then macrophages were fixed and stained with a cocktail of antibodies consisting of α-CD14-APC, α-CD64-APC and α-CD11b-APC. Macrophages (APC^+^) appear red in the image and apoptotic cells (Cy3^+^) appear yellow. On the left is a representative image from a single field from 384-well (20X objective) enlarged to show macrophages that have engulfed apoptotic cells. Image on the right showing macrophages co-cultured with live Jurkat cells. Scale is indicated by bar representing 50μM shown in lower left corner of each image.
(PPTX)

S2 Fig Staurosporine-induced apoptosis is detectable within three hours of treatment. Cells were treated with staurosporine at 0.4μM, 0.2μM and 1.0μM concentrations and untreated control (Live) cells were cultured in parallel for 18 hours (A) or 3 hours (B) at 37°C. Flow cytometric analysis was performed to assess accumulation of cell debris to indicate cell death and frequency of cells having active caspase 3 to indicate apoptosis. Results from the 18 hour cultures are shown in A; scatter profiles are shown in the top row as FSC-H vs SSC-H dot plots and caspase 3 staining at the corresponding condition is shown in the bottom row. No apoptosis is detectable in cells treated with low staurosporine concentration (0.4μM) in that no active caspase 3 is detectable and no change in scatter profile is evident as compared to untreated cells. Active caspase 3 is readily detectable in cells treated with either 0.2μM or 1.0μM staurosporine along with an accumulation of cell debris evident in the scatter profile dot plots indicating cells are dying. Caspase 3 stain and scatter plots are shown for ungated populations. Scatter plots for cells stained for active caspase 3 (shown in Fig 1c) treated with 1.0μM staurosporine for 3 hours compared to untreated (live) cells are shown in (B). Note the absence of cell debris in the staurosporine-treated cells.
(PPTX)

S3 Fig Optimizating incubation time for PI calculation based on presence of intact apoptotic cells within macrophages. Macrophages were incubated with apoptotic cells and cultures were harvested for calculation of PI at 10 minute intervals with the longest culture time being 40 minutes (A). Control cultures consisting of either untreated cells (Live cells) or cytochalasin D-treated macrophages (CytoD treated) were set up in parallel and analyzed after a 40 minute incubation time. The PI was calculated as described in materials and methods. Data shown as the average PI+/- STDev and are statistically significant by one way ANOVA with p<0.0001; Dunnett’s multiple comparisons test demonstrates the PI at each time point is statistically different than that for live cells. The difference between the PI for live cells vs cytochalasin D treated (CytoD) is not significant using this test. Data shown is representative of 3 experiments. (B) Apoptotic cell break down is seen with longer incubation which confounds PI calculation. As ingested apoptotic cells are broken down, staining appears as smaller spots within the macrophages (white arrows).
(PPTX)

S4 Fig The PI obtained using high content imaging is impacted by Gas6/MerTK interaction. Macrophages were co-cultured with apoptotic cells (AC) for 20 minutes in the presence of mouse Gas6 alone or with MerTK-Fc then PI was calculated as described in materials and methods. Controls were as follows: AC + IFN-γ, AC + IFN-γ + CNTO 360 (human IgG1), untreated Jurkat cells (Live). Apoptotic cells were also added in the presence of recombinant MFG-E8. Macrophages were identified using a 1:1:1 cocktail of α-CD14, α-CD11b and α-CD64 all APC labeled. Apoptotic cells were identified as pHrodo^+^ (in Cy3 channel). No DAPI staining was included. Analysis using one way ANOVA gives p < 0.005 indicating statistical significance. Tukey’s multiple comparisons test indicates statistically significant differences between groups as indicated by bars and asterisks on the graph. Data shown is from one of three studies.
(PPTX)

S1 Movie Macrophage in the center of the field (unstained) undergoes sequential phagocytosis of two presenting apoptotic Jurkat cells (pHrodo stained). Time lapse represents six frames over 30 minutes at 5 min intervals.
(MOV)
