# Supplementary figures and images for: Discovering Molecules That Regulate Efferocytosis Using Primary Human Macrophages and High Content Imaging
Source: PLoS One. 2015 Dec 16;10(12):e0145078. doi: 10.1371/journal.pone.0145078 (PMC4686065; doi:10.1371/journal.pone.0145078)

## Slide 1
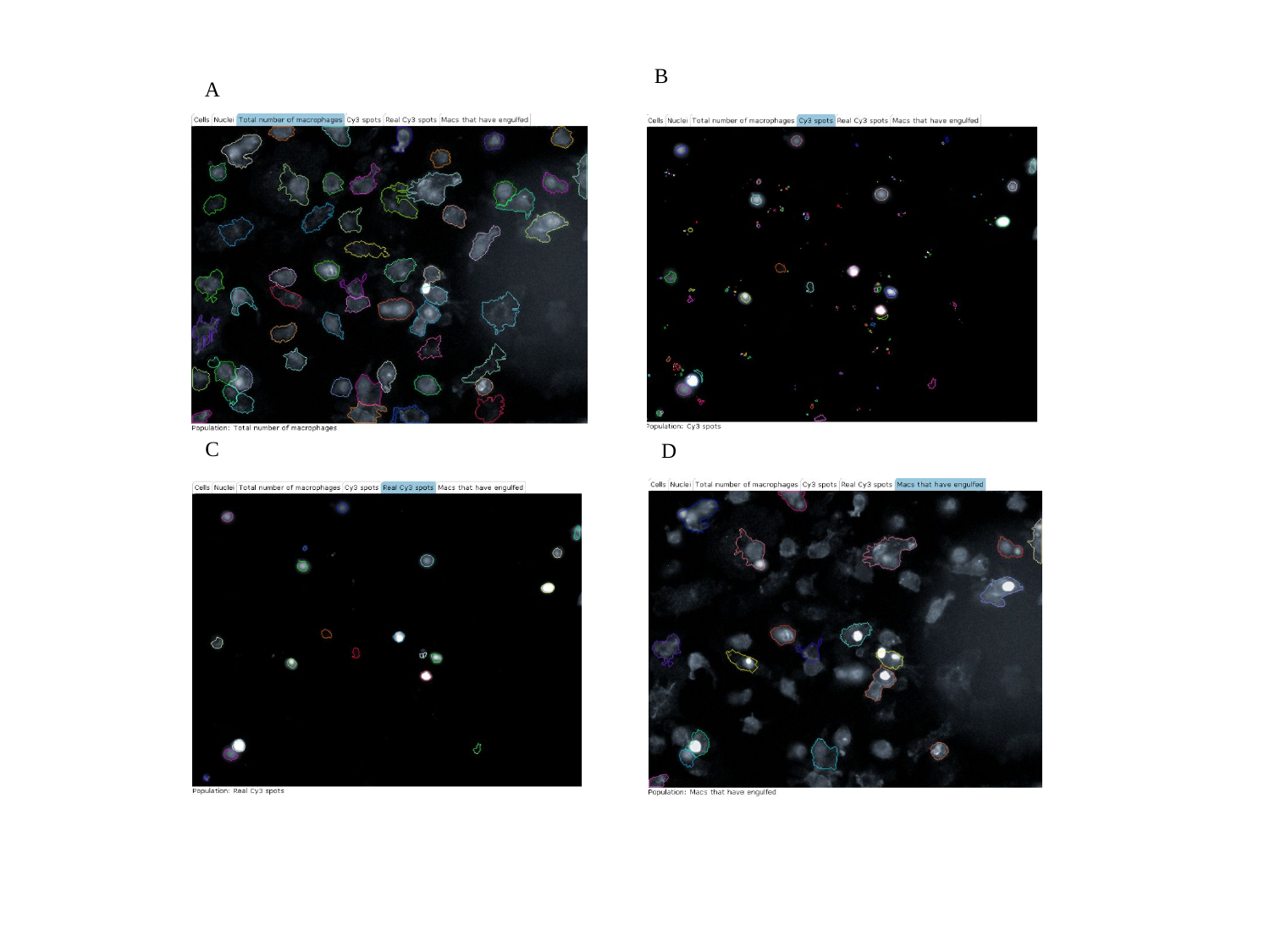

B
A
C
D

## Slide 2
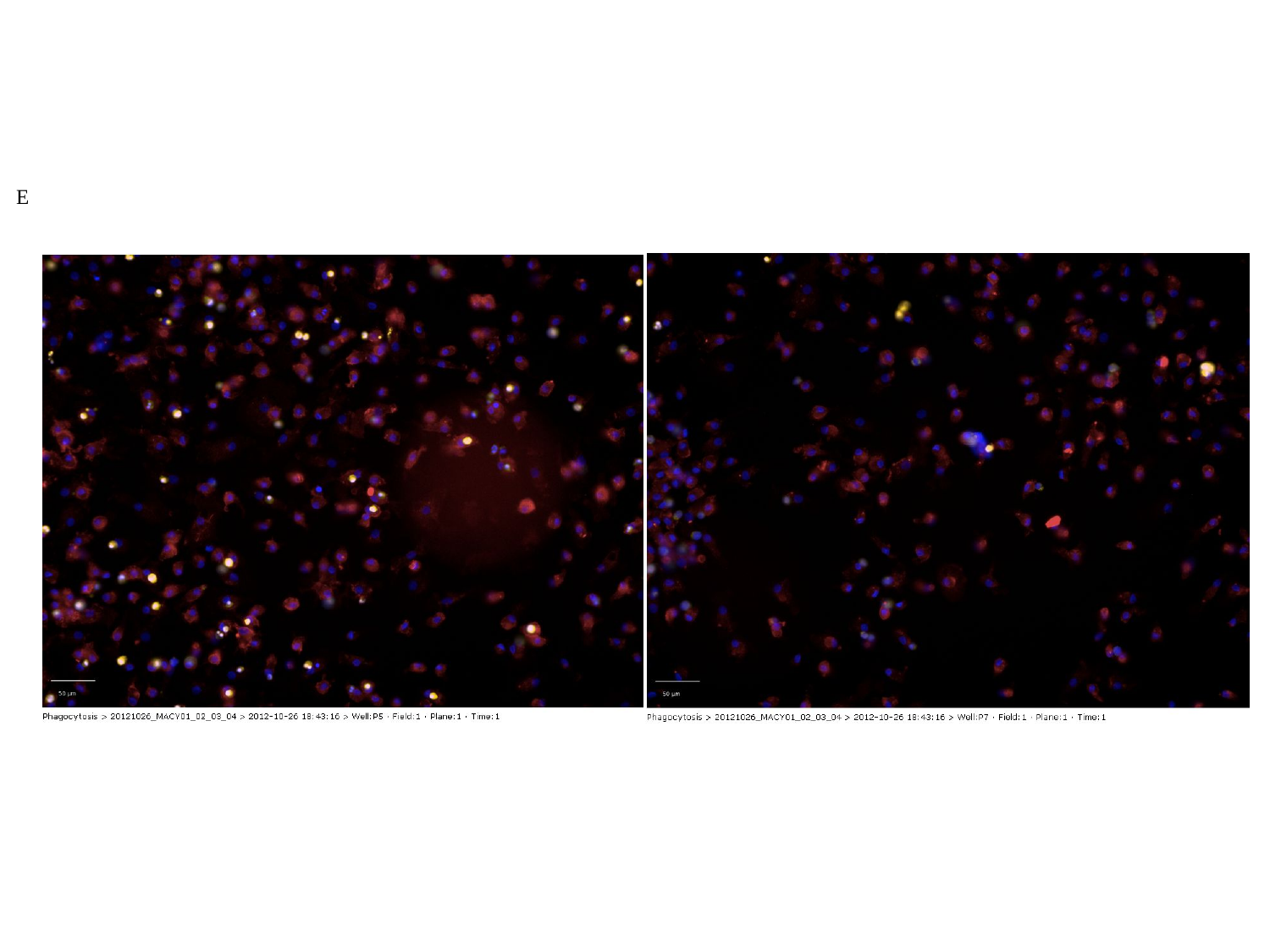

E

Supplement: S1 Fig — Analysis was performed using Columbus software and sample gates shown are as follows: (A) Find macrophages based on APC stain to define and select this population. (B, C) Define Cell region to minimize counting Jurkats not fully engulfed, find Jurkats based on pHrodo stain and define this population by size (Ingested Cells) and fluorescence intensity (Spots) and select this population (Spots). (D) Count macrophages as positive if they contain ≥ 1 spot/cell. (E) Intact apoptotic cells are detectable within macrophages. Macrophages were co-cultured with apoptotic and live Jurkat cells labeled with pHrodo dye for 30 minutes. Excess Jurkat cells were washed away then macrophages were fixed and stained with a cocktail of antibodies consisting of α-CD14-APC, α-CD64-APC and α-CD11b-APC. Macrophages (APC+) appear red in the image and apoptotic cells (Cy3+) appear yellow. On the left is a representative image from a single field from 384-well (20X objective) enlarged to show macrophages that have engulfed apoptotic cells. Image on the right showing macrophages co-cultured with live Jurkat cells. Scale is indicated by bar representing 50μM shown in lower left corner of each image. (PPTX) [file pone.0145078.s001.pptx]

## Slide 1
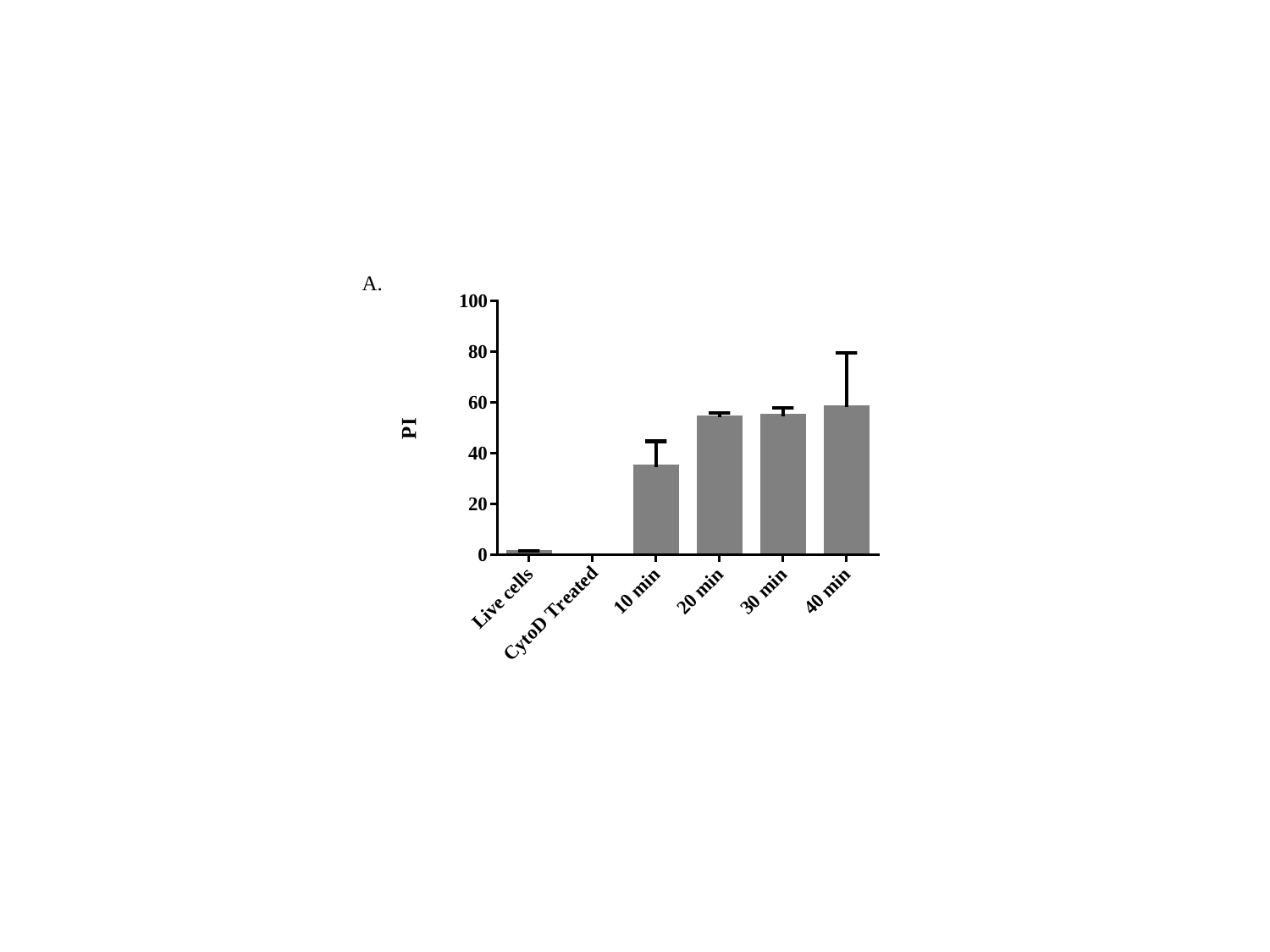

A.

## Slide 2
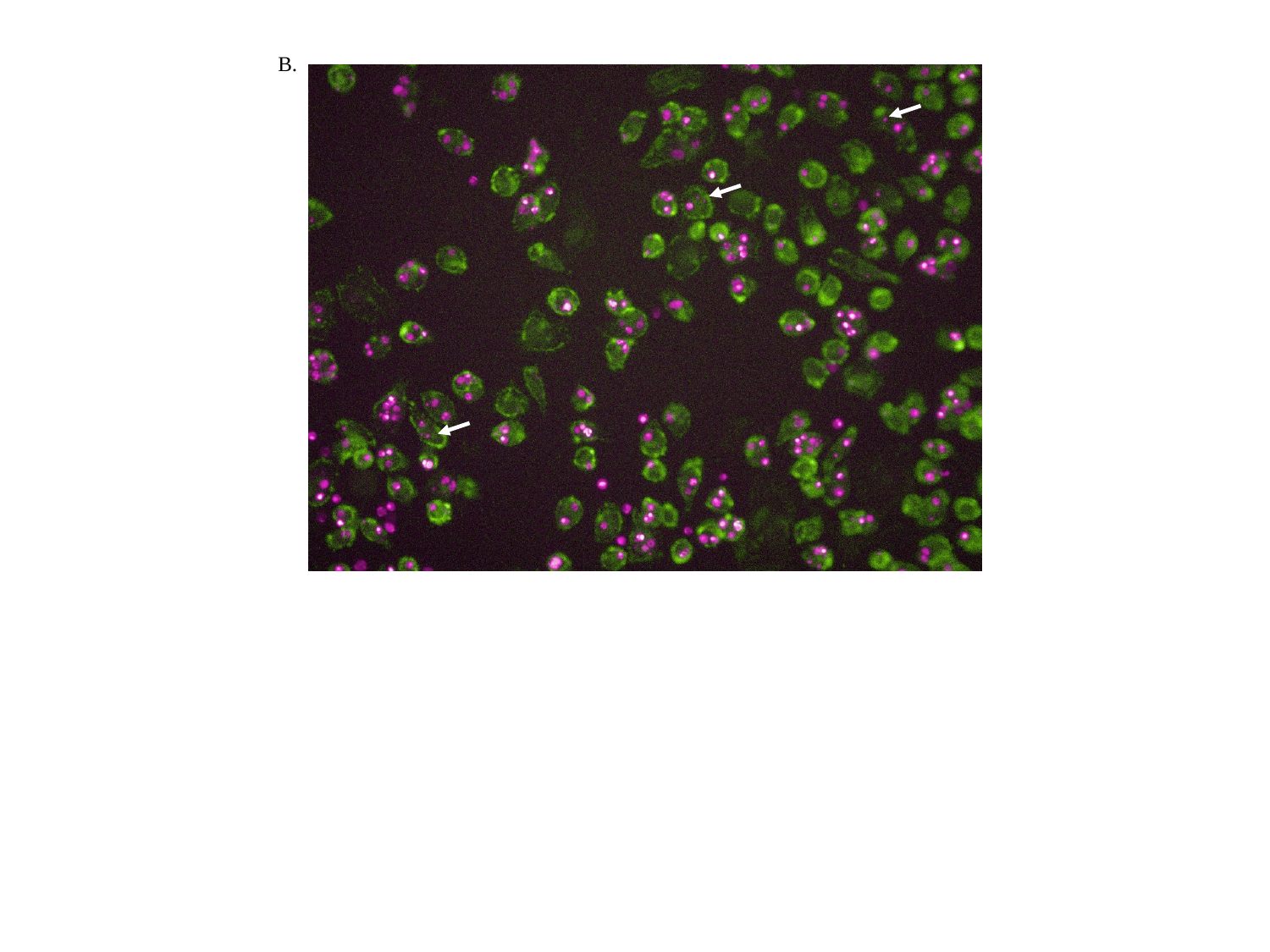

B.

Supplement: S3 Fig — Macrophages were incubated with apoptotic cells and cultures were harvested for calculation of PI at 10 minute intervals with the longest culture time being 40 minutes (A). Control cultures consisting of either untreated cells (Live cells) or cytochalasin D-treated macrophages (CytoD treated) were set up in parallel and analyzed after a 40 minute incubation time. The PI was calculated as described in materials and methods. Data shown as the average PI+/- STDev and are statistically significant by one way ANOVA with p<0.0001; Dunnett’s multiple comparisons test demonstrates the PI at each time point is statistically different than that for live cells. The difference between the PI for live cells vs cytochalasin D treated (CytoD) is not significant using this test. Data shown is representative of 3 experiments. (B) Apoptotic cell break down is seen with longer incubation which confounds PI calculation. As ingested apoptotic cells are broken down, staining appears as smaller spots within the macrophages (white arrows). (PPTX) [file pone.0145078.s003.pptx]

## Slide 1
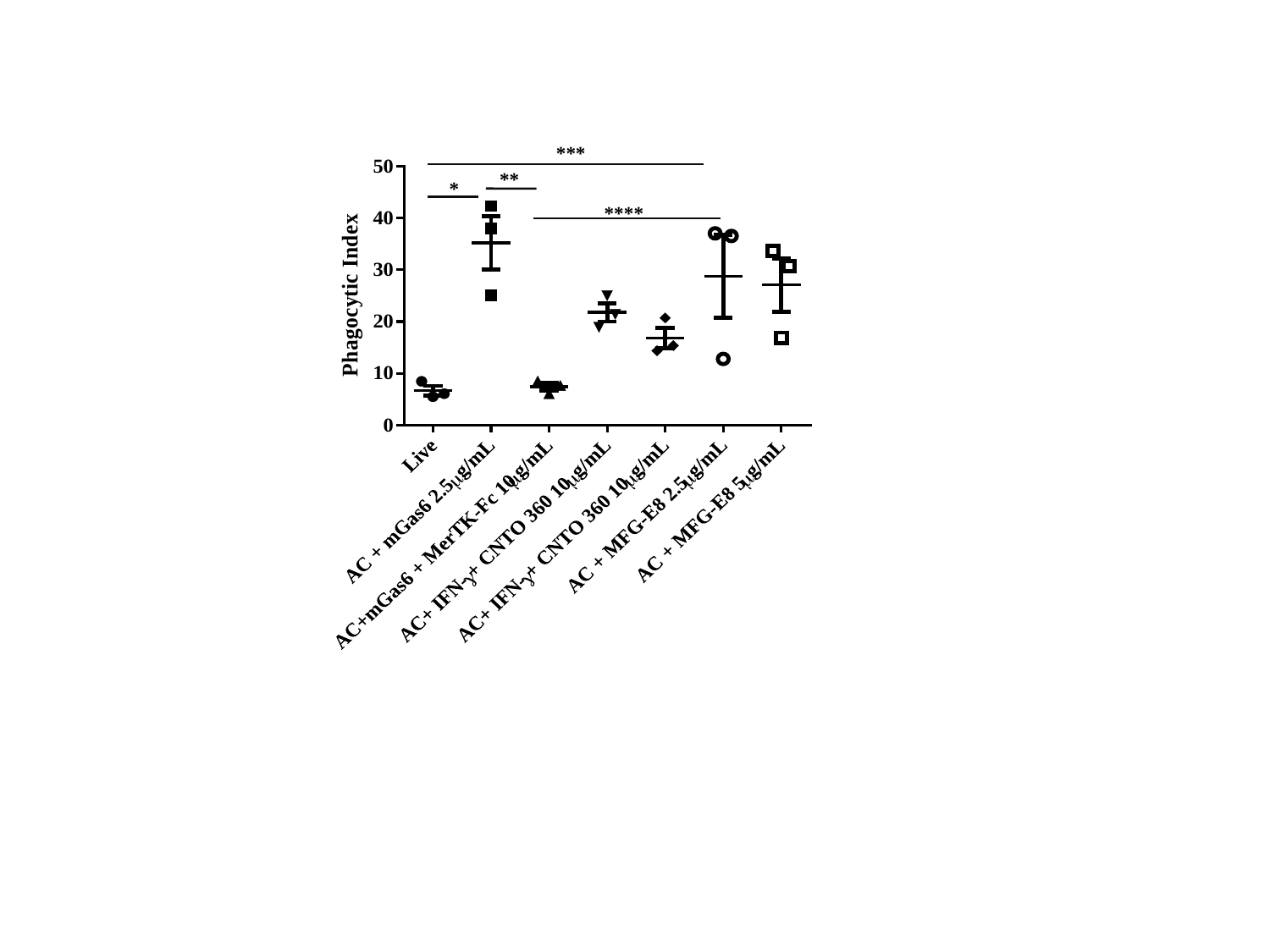

Supplement: S4 Fig — Macrophages were co-cultured with apoptotic cells (AC) for 20 minutes in the presence of mouse Gas6 alone or with MerTK-Fc then PI was calculated as described in materials and methods. Controls were as follows: AC + IFN-γ, AC + IFN-γ + CNTO 360 (human IgG1), untreated Jurkat cells (Live). Apoptotic cells were also added in the presence of recombinant MFG-E8. Macrophages were identified using a 1:1:1 cocktail of α-CD14, α-CD11b and α-CD64 all APC labeled. Apoptotic cells were identified as pHrodo+ (in Cy3 channel). No DAPI staining was included. Analysis using one way ANOVA gives p < 0.005 indicating statistical significance. Tukey’s multiple comparisons test indicates statistically significant differences between groups as indicated by bars and asterisks on the graph. Data shown is from one of three studies. (PPTX) [file pone.0145078.s004.pptx]
