# Supplementary material for: Discovering Molecules That Regulate Efferocytosis Using Primary Human Macrophages and High Content Imaging
Source: PLoS One. 2015 Dec 16;10(12):e0145078. doi: 10.1371/journal.pone.0145078 (PMC4686065; doi:10.1371/journal.pone.0145078)
Supplement: S2 Fig — Cells were treated with staurosporine at 0.4μM, 0.2μM and 1.0μM concentrations and untreated control (Live) cells were cultured in parallel for 18 hours (A) or 3 hours (B) at 37°C. Flow cytometric analysis was performed to assess accumulation of cell debris to indicate cell death and frequency of cells having active caspase 3 to indicate apoptosis. Results from the 18 hour cultures are shown in A; scatter profiles are shown in the top row as FSC-H vs SSC-H dot plots and caspase 3 staining at the corresponding condition is shown in the bottom row. No apoptosis is detectable in cells treated with low staurosporine concentration (0.4μM) in that no active caspase 3 is detectable and no change in scatter profile is evident as compared to untreated cells. Active caspase 3 is readily detectable in cells treated with either 0.2μM or 1.0μM staurosporine along with an accumulation of cell debris evident in the scatter profile dot plots indicating cells are dying. Caspase 3 stain and scatter plots are shown for ungated populations. Scatter plots for cells stained for active caspase 3 (shown in Fig 1c) treated with 1.0μM staurosporine for 3 hours compared to untreated (live) cells are shown in (B). Note the absence of cell debris in the staurosporine-treated cells. (PPTX) [file pone.0145078.s002.pptx]

## Slide 1
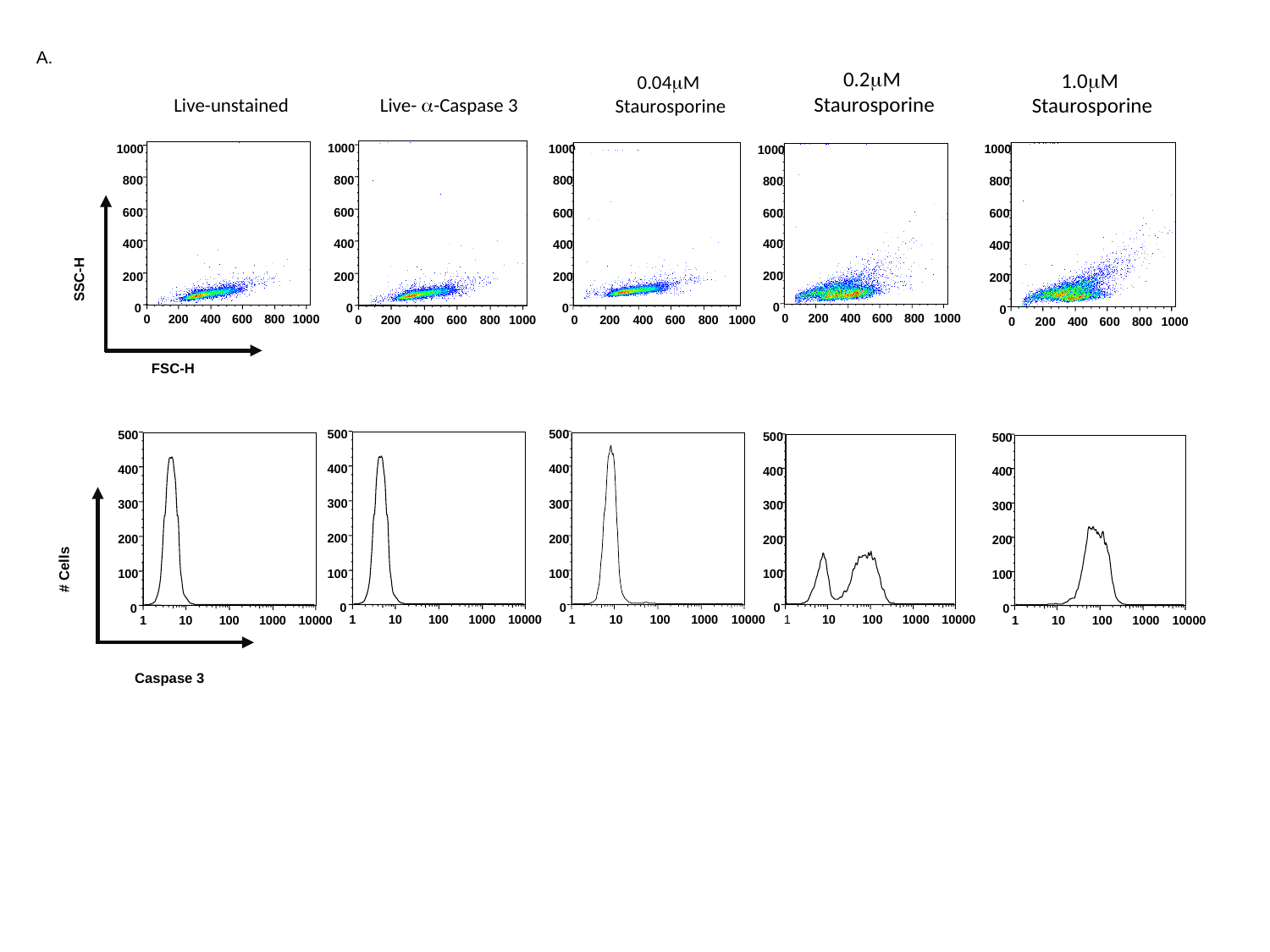

A.
0.2mM
Staurosporine
1.0mM
 Staurosporine
0.04mM
 Staurosporine
Live-unstained
Live- a-Caspase 3
1000
800
600
400
200
0
0
200
400
600
800
1000
1000
800
600
400
200
0
0
200
400
600
800
1000
1000
800
600
400
200
0
0
200
400
600
800
1000
1000
800
600
400
200
0
0
200
400
600
800
1000
1000
800
600
400
200
0
0
200
400
600
800
1000
SSC-H
FSC-H
500
400
300
200
100
0
1
10
100
1000
10000
500
400
300
200
100
0
1
10
100
1000
10000
500
400
300
200
100
0
1
10
100
1000
10000
500
400
300
200
100
0
1
10
100
1000
10000
500
400
300
200
100
0
1
10
100
1000
10000
# Cells
Caspase 3

## Slide 2
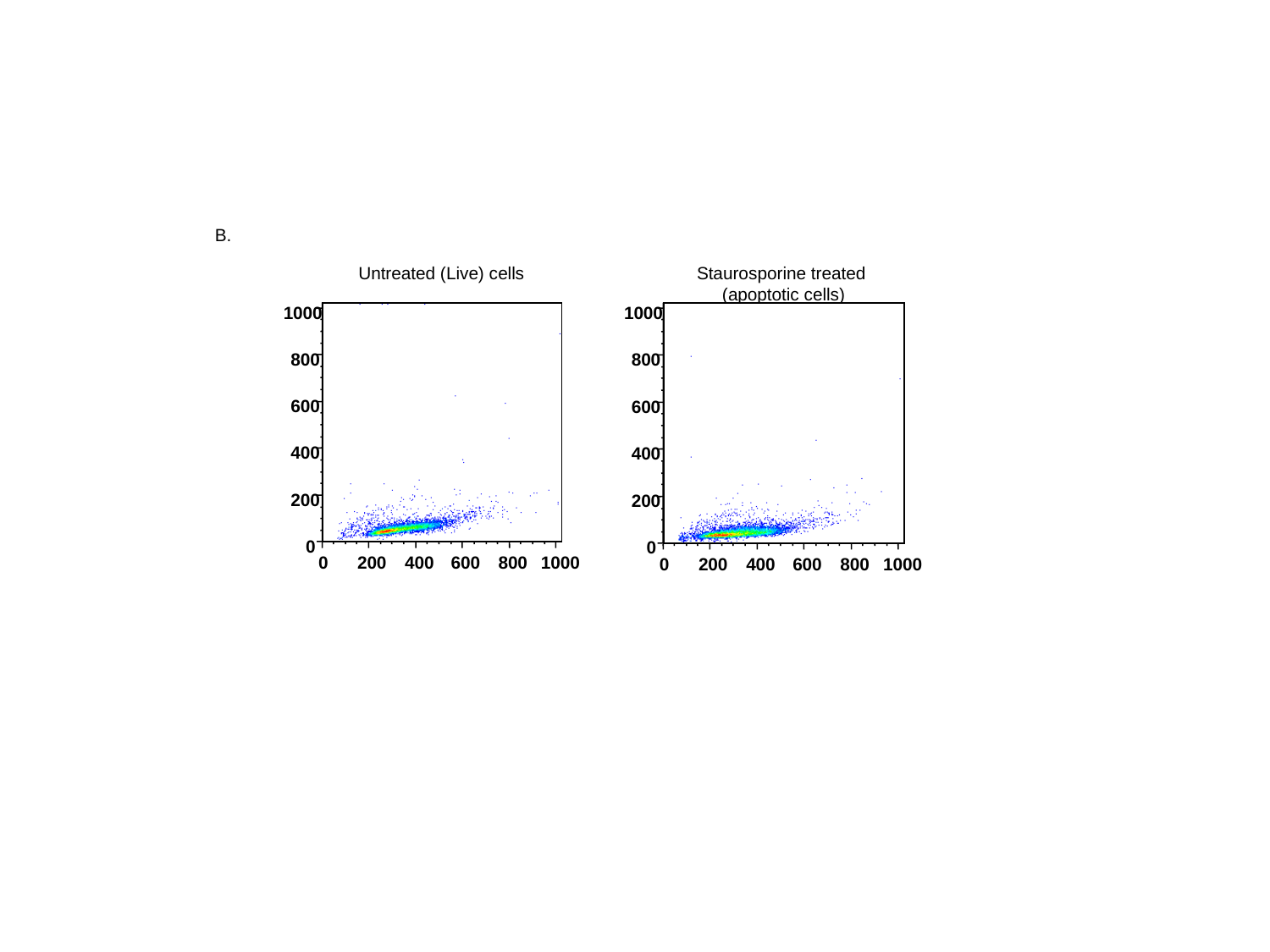

B.
Untreated (Live) cells
Staurosporine treated
 (apoptotic cells)
1000
800
600
400
200
0
0
200
400
600
800
1000
1000
800
600
400
200
0
0
200
400
600
800
1000
